# Hyperbaric oxygen therapy in combination with systemic treatment of sickle cell disease presenting as central retinal artery occlusion: a case report

**DOI:** 10.1186/1752-1947-8-370

**Published:** 2014-11-17

**Authors:** Handan Canan, Burak Ulas, Rana Altan-Yaycioglu

**Affiliations:** 1 Department of Ophthalmology, Baskent University School of Medicine, Dadaloglu mah, Serinevler 2591 No 4/A 01250, Yuregır, Adana, Turkey

**Keywords:** Hyperbaric Oxygen Therapy, Retinal Artery Occlusion, Sickle Cell Disease

## Abstract

**Introduction:**

We describe hyperbaric oxygen therapy for the treatment of central retinal artery occlusion in a young adult with sickle cell disease.

**Case presentation:**

A 25-year-old Turkish man with a history of sickle cell disease developed sudden painless loss of vision in the left eye and was hospitalized for diagnosis and treatment. Central retinal artery occlusion was diagnosed with retinal whitening, cherry red spot, and delayed arteriovenous transit on fluorescein angiography. He underwent exchange transfusion and hyperbaric oxygen therapy. In the following three months, his visual acuity improved to 20/30.

**Conclusions:**

In this present case with sickle cell disease, the visual acuity improved with hyperbaric oxygen therapy in addition to systemic therapy. The result of our case suggests that hyperbaric oxygen therapy may be beneficial in the treatment of central retinal artery occlusion.

## Introduction

Central retinal artery occlusion (CRAO) is not a frequent cause of blindness. The visual acuity loss is sudden and painless, and is often severe and permanent in CRAO. The incidence of CRAO in young people is less than 1:50,000 [1]. Several conditions are reported to be associated with CRAO [1]. The majority of occlusive conditions result in infarction of the inner retina [2]. Sickle cell disease (SCD) is one of the diseases resulting in vaso-occlusion. It presents in two forms, with non-proliferative retinopathy being the most common ophthalmic manifestation. Ophthalmic complications associated with visual loss are usually characterized by proliferative retinopathy [3,4] and CRAO is a rare complication of this disease. The treatment and prognosis of CRAO in SCD is still not certain and there are no guidelines for CRAO in SCD in the literature.

We report a case of CRAO due to SCD and report the visual recovery with hyperbaric oxygen (HBO) and systemic treatment.

## Case presentation

**Figure 1:**
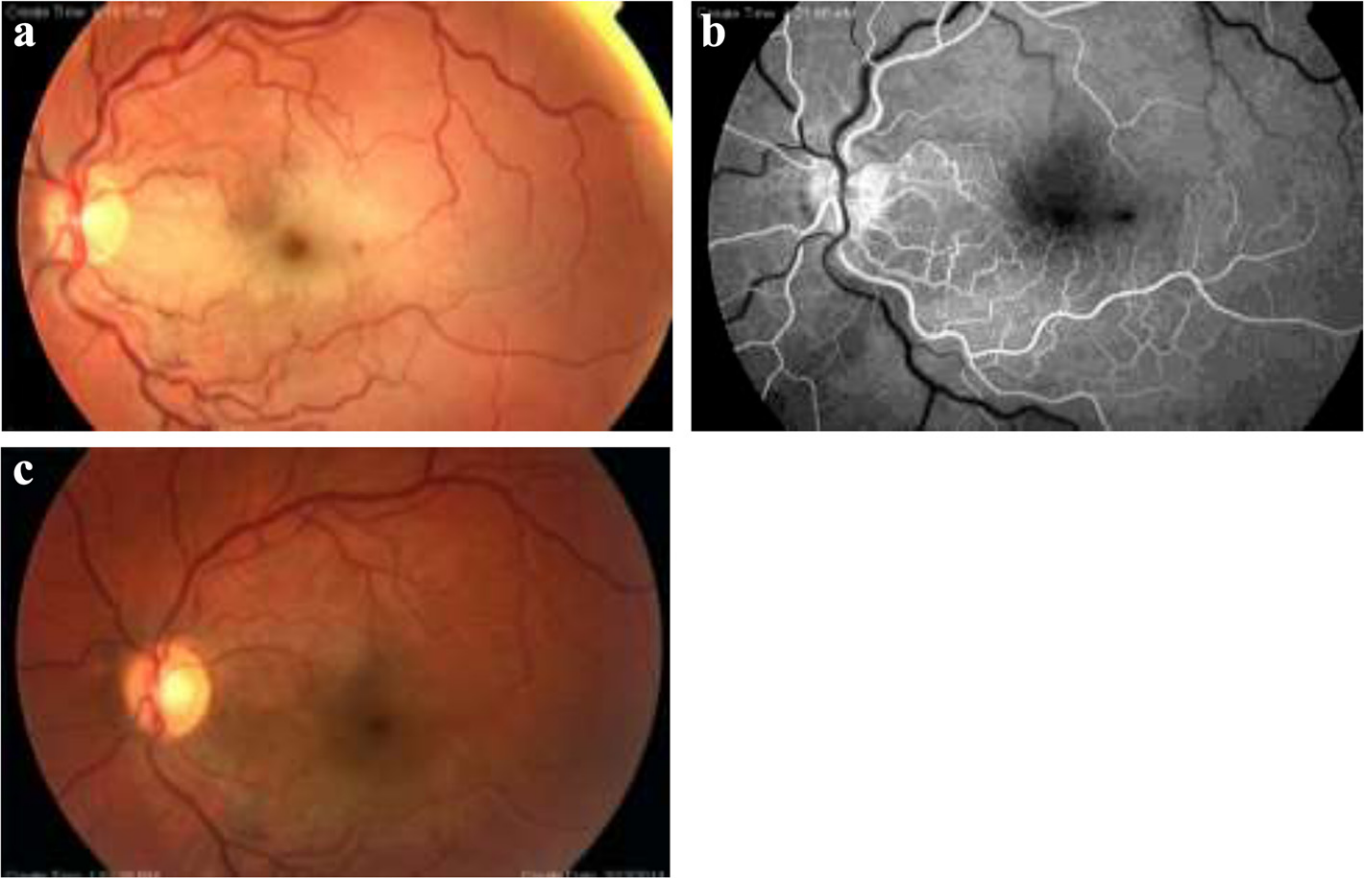
**Fundus examination of central retinal artery occlusion in sickle cell disease.** CFP (colored fundus photograph) at initial assessment **(a)** shows central retinal artery occlusion with pale, thickened retinal infarction, a dot hemorrhage inferotemporal to the fovea, and cherry red spot in the left eye. The early-phase of fluorescein angiography **(b)** the delay in arteriovenous transit time at 21 seconds and ischemia in the macula was discernible. CFP at his six-month visit showing the disappearance of whitening and cherry-red spot **(c)**.

**Figure 2:**
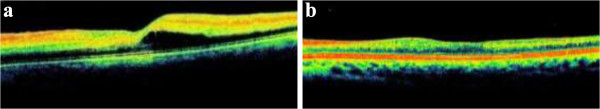
**Optical coherence tomography (OCT) scan of central retinal artery occlusion in sickle cell disease.** OCT of left eye shows macular edema (CFT, 403μm) at presentation **(a)**. At his six-month visit, the macula was normal (CFT, 200μm) **(b)**.

A 25-year-old Turkish Caucasian man with homozygous (Hb SS) SCD presented to our emergency department with a sudden, painless loss of vision in the left eye for the preceding two hours. His best-corrected visual acuity (BCVA) was 20/20 in the right eye and counting fingers in the left eye. Relative afferent pupillary defect (RAPD) was present in the left eye. His anterior segment examination was normal bilaterally and his intraocular pressure was 21mmHg in both eyes, as measured by Goldmann applanation tonometry. Fundus examination revealed a pale and thickened retina, suggesting retinal edema centered on the papillomacular bundle, in addition to increased vascular tortuosity in the left eye (Figure 1a). His right fundus was normal. A fundus fluorescein angiography (Figure 1b) showed lack of arterial filling of the artery, delayed arteriovenous transit time at 21 seconds, and peripheral capillary non-perfusion in the left eye. His retinal vasculature was normal in the right eye. An optical coherence tomography showed increased central foveal thickness (CFT) and inner retinal layer thickening in the left eye. The CFT at baseline was 403μm (Figure 2a). He was subsequently diagnosed with left CRAO.

He was hospitalized immediately with the aim of decreasing the blood viscosity. To restore vascular perfusion, hematologists started emergent exchange transfusion within three hours following admission. His BCVA did not change immediately after transfusion. Thus, in addition to the systemic therapy, a HBO therapy was planned, and started within the first 24 hours of admission. The treatment protocol was 2.5 ATA (atmosphere absolute) for 120 minutes twice daily for the first seven days, and then once a day for the following six days, for a maximum total of 20 sessions. An improvement in BCVA to 20/200 was observed after two sessions (24 hours), and to 20/60 at the end of 20 sessions. At the three-month visit his BCVA had increased to 20/30, and remained so until his last visit at six months. At the last visit his fundus examination showed the recovery of retinal ischemia (Figure 1c) and OCT scan showed CFT to be 200μm (Figure 2b).

## Discussion

Sickle cell disease is a hereditary blood disorder characterized by red blood cells that assume an abnormal, rigid, sickle shape. Sickling decreases the blood cells' flexibility and results in a risk of various life-threatening complications including vaso-occlusion. The sickle syndromes have the highest incidence in black Africans and African-Americans, but are also being found in people from Mediterranean countries [4]. Almost any organ can be affected by this vasoocclusive phenomenon. In retina, the sickled red blood cells obstruct the retinal vessels leading to reduced blood flow and retinal vascular occlusion. There are only a few published reports of retinal artery occlusion associated with SCD [5-7]. Treatment experience of CRAO with SCD is even more limited. In these cases, exchange transfusion should be considered first in order to restore vascular perfusion [6]. Hydroxyurea may be recommended for prophylactic treatment to prevent systemic crises [5]. The alternatives for the treatment of CRAO in SCD consist of pharmacological and mechanical reduction of ocular pressure, HBO therapy, ocular massage, and direct thrombolysis, however their benefits are not well proven [6]. HBO therapy was reported to be useful in the treatment of CRAO [8-11]. This therapy shows its effect by increasing tissue oxygenation. Also, in our case reperfusion of the retina was observed following HBO therapy. This suggests that increased arterial pO2 may reverse sickling of erythrocytes in the microcirculation of the retina and optic nerve. Further, the elevated level of pO2 in tissue may diminish the risk of tissue ischemia and retinal infarction [12]. The limitation of this case report is that we cannot be certain of how much of the improvement in visual acuity may be attributed to HBO therapy or to the exchange therapy. A recent study of Menzel-Severing *et al*. reported that in the treatment of cases with CRAO, combined HBO and hemodilution treatment achieved more improvement of visual acuity compared to hemodilution alone [13].

In this case we report the benefit of HBO therapy in addition to systemic treatment in a young adult with SCD and CRAO. To the best of our knowledge this is the first case in the current literature to report the use of HBO in CRAO in a case with SCD. Our case suggests that even in cases with SCD, HBO therapy may be a valuable treatment alternative, in addition to the systemic treatment in CRAO.

## Conclusions

The result of our case suggests that HBO therapy may be beneficial in the treatment of CRAO. Therefore, we recommend that HBO therapy should be applied as soon as possible in cases with CRAO and SCD, in addition to exchange transfusion, in order to prevent irreversible damage from complete occlusion. A few case reports, of course, are not adequate to decide on whether HBO therapy should be the treatment of choice in this condition. Further research to document the safety and efficacy of HBO therapy in SCD is necessary.

## Consent

Written informed consent was obtained from the patient for publication of this case report and any accompanying images. A copy of the written consent is available for review by the Editor-in-Chief of this journal.

## Abbreviations

BCVA: Best corrected visual acuity; CFT: Central foveal thickness; CRAO: Central retinal artery occlusion; CFP: Colored fundus photograph; HBO: Hyperbaric oxygen therapy; OCT: Optical coherence tomography; SCD: Sickle cell disease.

## Competing interests

The authors declare that they have no competing interests.

## Authors’ contributions

HC was the major contributor to writing the manuscript. BU drafted the manuscript. RAY helped with the drafting and critical review of the manuscript. All authors read and approved the final manuscript.
